# Determination of Local Strain Distribution at the Level of the Constituents of Particle Reinforced Composite: An Experimental and Numerical Study

**DOI:** 10.3390/ma13173889

**Published:** 2020-09-03

**Authors:** Witold Ogierman, Grzegorz Kokot

**Affiliations:** Department of Computational Mechanics and Engineering, Faculty of Mechanical Engineering, Silesian University of Technology, Konarskiego 18a Street, 44-100 Gliwice, Poland; grzegorz.kokot@polsl.pl

**Keywords:** particle-reinforcement, strain transfer, micromechanics, mechanical properties

## Abstract

This paper is devoted to numerical and experimental investigation of the strain field at the level of the constituents of two-phase particle reinforced composite. The research aims to compare the strain distributions obtained experimentally with the results obtained by using a computational model based on the concept of the representative volume element. A digital image correlation method has been used for experimental determination of full-field strain. The numerical investigation was conducted by the finite element analysis of the representative volume element. Moreover, usage of the novel method of assessment of the speckle pattern applicability for the measurement of local fields by using the digital image correlation method has been proposed. In general, the obtained experimental and numerical results are in good agreement although some discrepancies between the results have been noticed and discussed.

## 1. Introduction

The local strain and stress fields at the level of the constituents of composite materials are inhomogeneous due to contrast in the mechanical properties of matrix and reinforcement. The strain and stress fields depend on several factors like the shape, volume fraction and spatial distribution of the reinforcement, and also the interface between the phases. The fundamental strategy in continuum micromechanics is the analysis of the behavior of the representative volume element (RVE), which statistically represents the real microstructure of the material. The RVE can be generated in a way to model the real microstructure very closely, for example by basing on the microtomography scans [1,2,3,4]. However, such detailed description of the microstructure may lead to a prohibitive computation time, particularly in the case of multi-scale computations. Therefore, very often, the RVE is considered as a model for simplifying the geometrical features of the real microstructure to provide a reasonable computation time [5,6]. Various numerical methods can be used for the analysis of the RVE, like, for instance, the boundary element method (BEM) [7,8,9,10], or the fast Fourier transform-based method (FFT) [11,12,13]. However, the most frequently used is the finite element method (FEM), which has become a standard approach. The literature presents numerous works that use the FEM for evaluation of the stress and strain fields at the level of the constituents [14,15,16,17,18,19]. On the other hand, there is a limited number of studies devoted to the experimental determination of local fields that allows for the validation of numerical models. In this case, the digital image correlation (DIC) can be applied, since it is a non-contact method and has got no inherent length scale. It is widely used in the determination of the behavior of composite materials at the macroscopic level [20,21,22,23,24,25,26,27], although several recent studies have reported on the application of the DIC in measuring the local strain fields. Berfield et al. [28] used the DIC method for the determination of displacement fields near isolated silica microspheres embedded in polymer materials. The works of Canal et al. [29] and Mehdikhani et al. [30] present the application of the DIC for the analysis of displacement and strain fields in long fiber-reinforced composites. Mehdikhani et al., in another work [31], performed analysis for detection and quantification of matrix cracks in carbon fiber composite laminates employing the DIC, and another study related to laminates was conducted by Orell et al. [32]. Koohbor et al. [33] and Anzelotti et al. [34] applied the DIC for analysis of the meso-scale strain localization of a woven reinforced composite. In the case of the particle-reinforced composites, which are the subject of the present work, experimental results presenting deformation behavior at the level of constituents can be found in the work of Hadjem-Hamouche et al. [35]. There are also several interesting papers associated with measuring the local strain fields by the DIC related to steels [36,37,38,39].

The present paper is devoted to both numerical and experimental determination of the local strain fields at the level of constituents. For numerical modelling, a standard approach based on the Finite Element Analysis (FEA) of the RVE containing particles with idealized geometry was used. The main aim of this paper is to compare the strain field distributions obtained using the simplified numerical model with the full-field strain measured experimentally by using the DIC. Another goal of this paper is to develop a novel approach for assessing speckle pattern applicability for the measurement of local fields at the level of constituents of the composite. One of the most important factors impacting the accuracy of the strain measurements by using the DIC method is the quality of the speckle pattern. In particular, when the analysis is performed on the level of composite constituents and high strain heterogeneity is expected, unacceptable errors related to the DIC analysis can be made. Moreover, even if the speckle pattern possesses appropriate parameters, it can lead to wrong results when its size is too big in comparison to the characteristic length of the microstructure (for example, the size of the reinforcing particles). In this case, the strain field may be formally correctly determined; however, the local strain concentration due to reinforcing particles can be unexposed (averaged). Therefore, during this work, an original method for assessing the DIC measurement accuracy was applied. The conception of the method is based on the generation of images that represent the artificially deformed specimen with respect to the results of the reference FE simulation related to the microgeometry behind the speckle pattern. This concept was proposed in the previous work of authors [40]; however, it was associated with artificially generated speckle patterns by prescribing a random grayscale level of pixels. This paper concentrates on a real specked pattern that was used in DIC analysis at the level of constituents of the composite.

## 2. Materials and Methods

### 2.1. Method of Assessment of the Speckle Pattern

**Figure 1:**
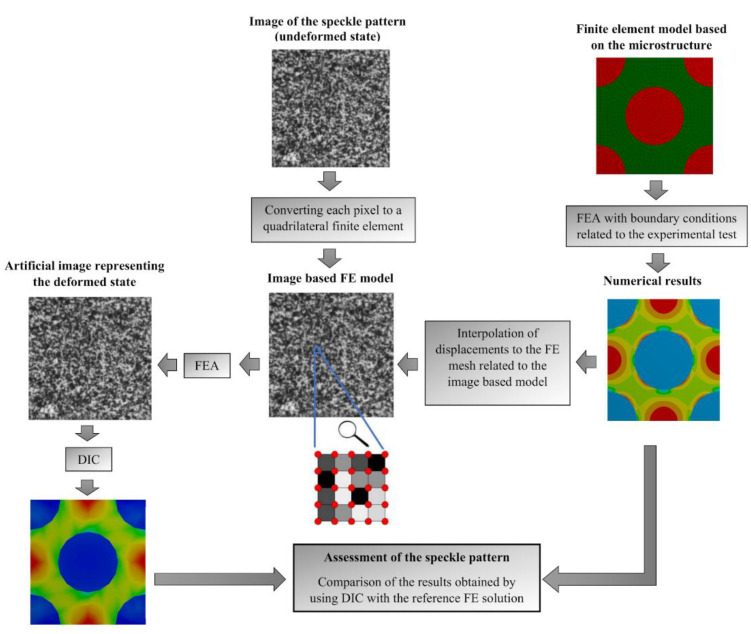
Flowchart of the method of the DIC measurement accuracy.

A new method of assessment of the DIC measurement accuracy has been proposed to investigate if the created speckle pattern is appropriate for exposing the local strain field heterogeneity at the level of the constituents. The idea of the method is based on the generation of the virtually deformed image referencing to the original image of the speckle pattern. A flowchart illustrating the proposed method is presented in Figure 1. The input data to the procedure is associated with the image of the speckle pattern created on the surface of the tested specimen and the FE model based on the microgeometry. It has to be underlined that purpose of the FE model, within this procedure, is not to approximate the behavior of a real tested material as close as possible but rather to provide the prediction on how the strain may be distributed for the tested composite. In general, the microgeometry can be reconstructed directly from the microscopic image or by using the microtomography. However, for the sake of simplicity, it can be created virtually in a way to be statistically equivalent to the real, which should be much more convenient in application. Boundary conditions should be applied in a way to model the real conditions of the experimental, for example when the specimen undergoes compression test the uniaxial compressive strain has to be applied to the FE model. The next step is the generation of the image-based FE model with respect to the image of the speckle pattern. In this case, the pixels from the image are converted to quadrilateral finite elements. Then the displacements which are the result of the FEA based on the microgeometry are interpolated to the FE mesh related to the image-based model. Afterwards, the FEA of the image-based model is performed with the interpolated displacements as the boundary condition. The result of the analysis is the artificial image representing the deformed state which is, together with the original image, an input data to the DIC analysis. It has to be underlined that the accuracy of the DIC depends not only on the speckle pattern itself but also on the subset size (facet size) which has to be appropriately adjusted. Finally, the result of the DIC analysis can be compared with the reference “accurate” FEM solution. This is done by using the developed original procedures enabling the DIC results to be transferred directly to the FEM system.

### 2.2. Experimental Test

**Figure 2:**
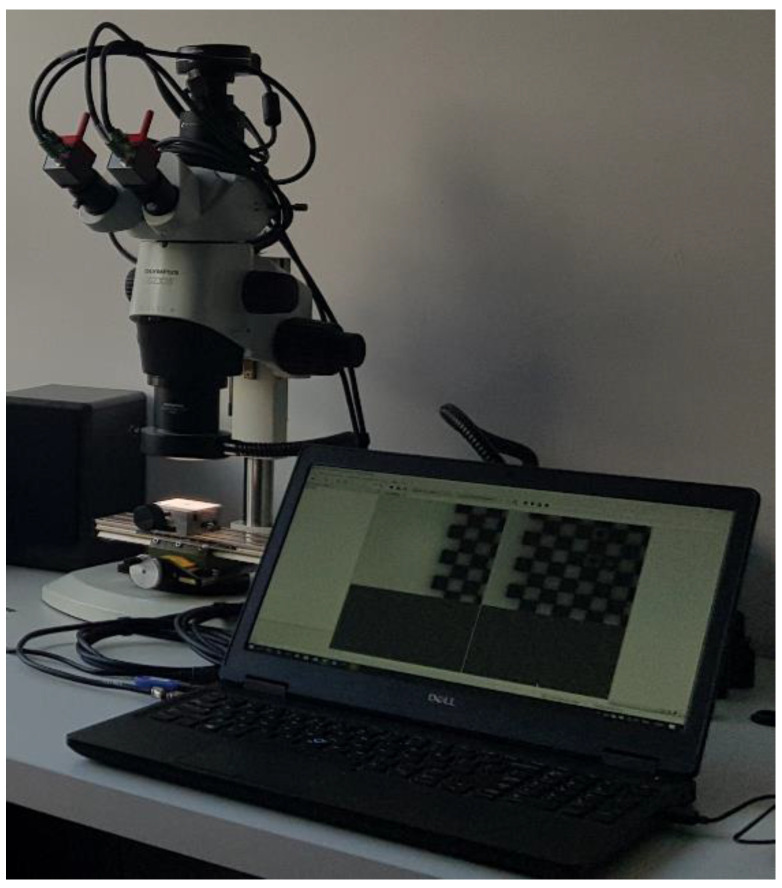
Equipment used for capturing the images of the tested specimen, and DIC analysis.

**Figure 3:**
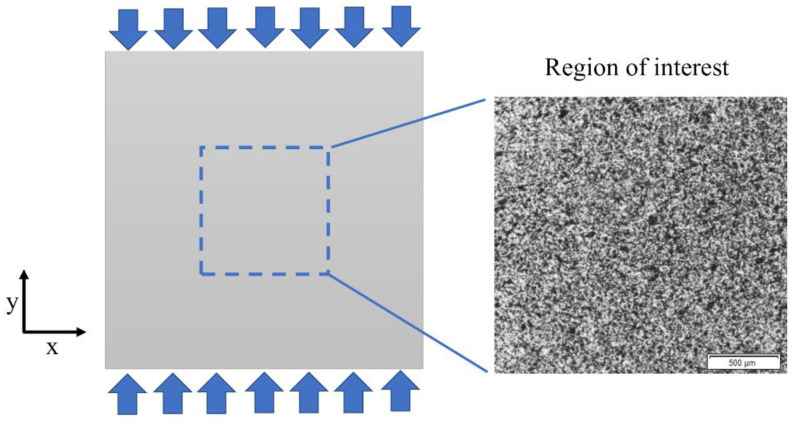
Test overview with detailed view on the speckle pattern in the region of interest.

**Figure 4:**
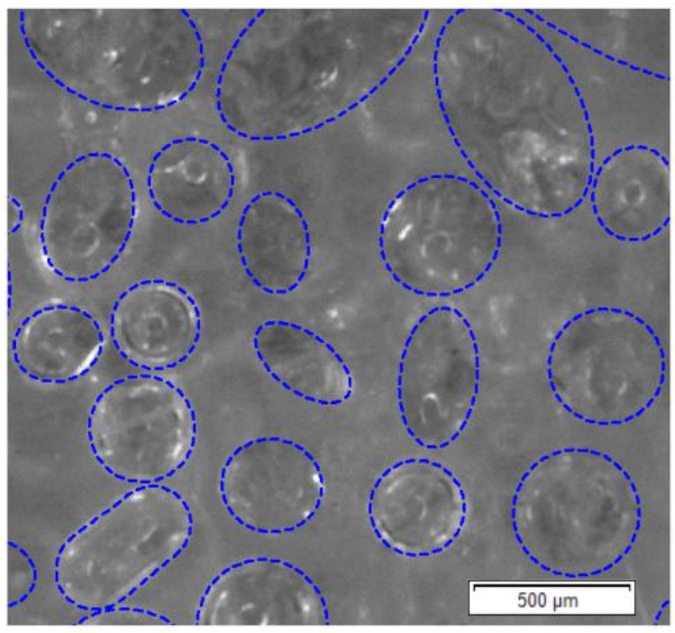
The reinforcing particles in the region of interest behind the speckle pattern.

Experimental determination of full-field strain was carried out by using the DIC method. Olympus SZX16 optical microscope (Olympus Corp., Tokyo, Japan) (Figure 2) was applied for observation of the specimen microstructure and capturing the images during the deformation. A material which was used in the investigation is epoxy resin reinforced with glass particles. Cubic specimen of the edge length of 10 mm was prepared, the surface intended for the strain measurement was polished and then the speckle pattern was created by using the fine point airbrush Infinity CR plus (Harder & Steenbeck, Norderstedt, Germany). The specimen was placed in the loading unit which is combined with the microscope and allows for compression of the specimens. A scheme presenting the test setup and the region of interest with detailed view on the speckle pattern is presented in Figure 3. The microscopic image that visualize the reinforcing particles embedded in the matrix placed in the region of interest is presented in Figure 4. The images representing an undeformed and deformed state of the specimen have been collected and then processed for full-field strain and displacement analysis by using DIC method (Istra 4D v4.6.1.374 software, Dantec Dynamics). The correlation process for the selected region of interest was conducted by taking the following parameters: facet size—21 pixels, grid spacing—20 pixels. Those parameters were taken as the optimal according to DIC measurements practice guide [41]. Finally, the obtained DIC results were transferred to ANSYS 18.2 software for the purpose of convenient comparison with the results of FEA based on the RVE.

### 2.3. Numerical Modeling

**Figure 5:**
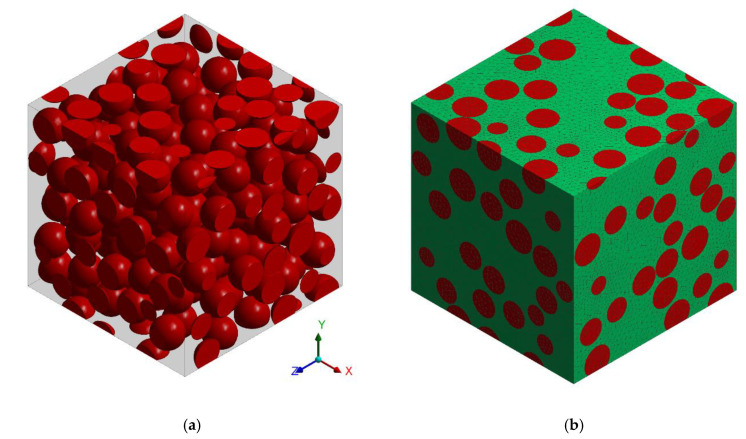
The representative volume element (RVE): (**a**) geometrical model; (**b**) finite element mesh.

The numerical analysis of the particle reinforced composite is performed in standard fashion associated with the FEA of the RVE. A cubic RVE representing statistically the microstructure of particle reinforced composite has been generated by using Digimat 5.1.1 software (MSC Software, Newport Beach, CA, USA). The RVE consists of approximately 200 nonintersecting spherical particles of the same size which spatial distribution was determined in a random way (Figure 5a). Usage of the proposed simplified model of the microstructure is justified by the fact that in case of the tested composite the reinforcing particles have shape varying from spherical to ellipsoidal with relatively low aspect ratio, the particles are randomly oriented and have smooth surfaces. ANSYS software was applied for building the numerical model of the RVE, created finite element mesh consisting of tetrahedral elements with quadratic shape functions is presented in Figure 5b. Periodic boundary conditions [42,43] which enforce the uniaxial compressive strain have been applied.

## 3. Results and Discussion

### 3.1. Testing of the Speckle Pattern

**Figure 6:**
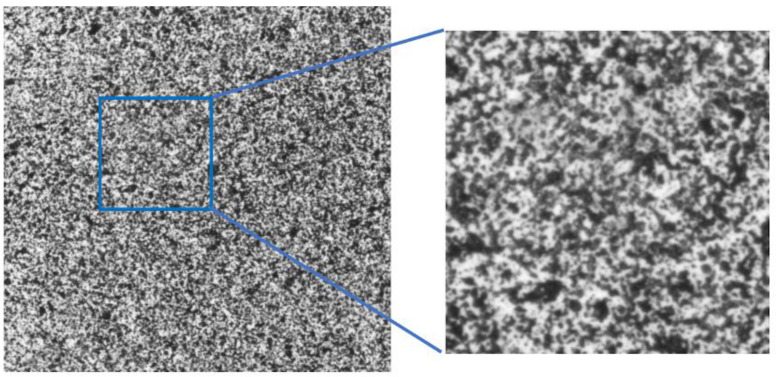
Fragment of the speckle pattern that was tested.

**Figure 7:**
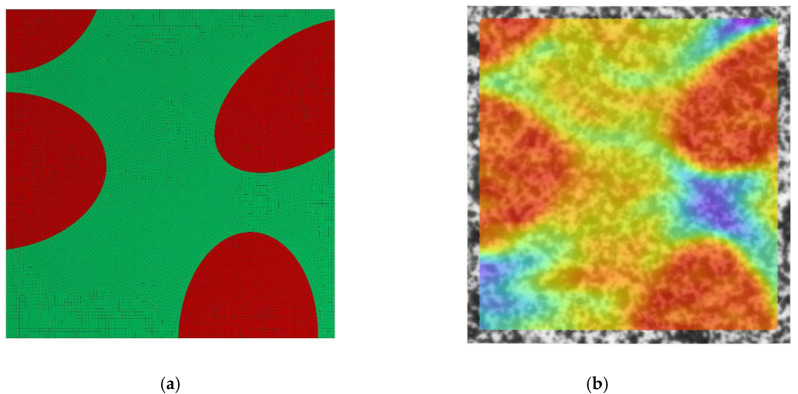
Model of the fragment of the microstructure that is expected behind the speckle pattern: (**a**) FE model, (**b**) overview on the result of the DIC analysis in Istra 4D software (ε_yy_ distribution).

**Figure 8:**
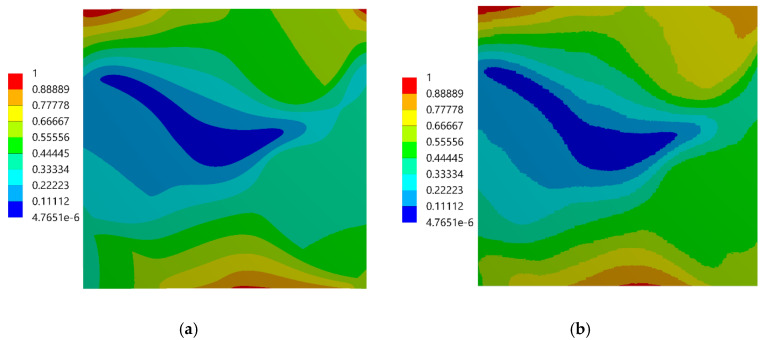
Distribution of the total normalized displacement: (**a**) FE reference solution, (**b**) DIC analysis.

**Figure 9:**
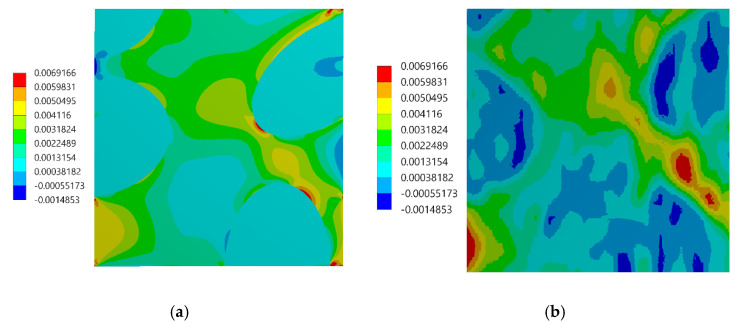
Distribution of the ε_xx_ strain: (**a**) FE reference solution, (**b**) DIC analysis.

**Figure 10:**
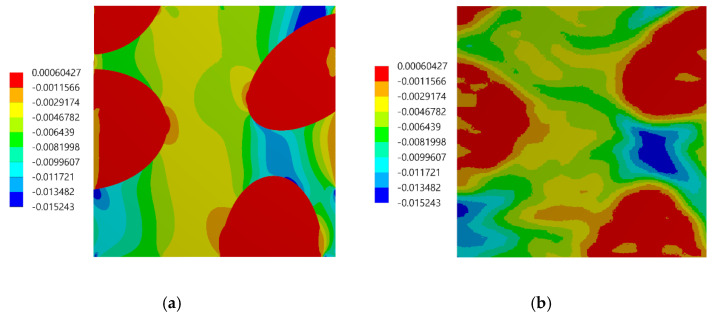
Distribution of the ε_yy_ strain: (**a**) FE reference solution, (**b**) DIC analysis.

**Figure 11:**
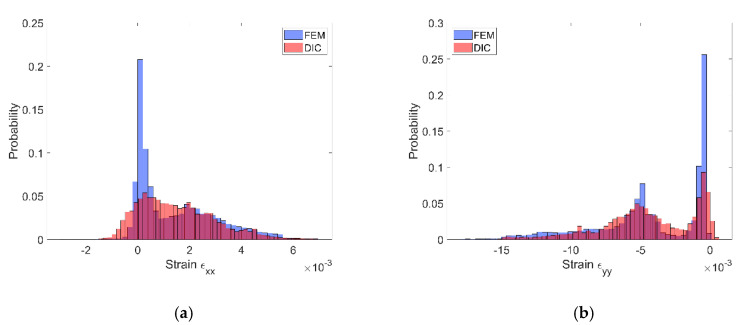
Histograms representing the strain distributions determined by DIC and FE reference solution: (**a**) ε_xx_ component, (**b**) ε_yy_ component.

The assessment of the DIC measurement accuracy was performed on the fragment of the speckle pattern presented in Figure 6 by using the procedure described in Section 2.1. Figure 7a presents a simplified two-dimensional (plane strain) FE model representing a fragment of the composite’s microstructure associated with the tested fragment of the speckle pattern. Periodic boundary conditions enforcing the uniaxial compressive strain have been applied. Linear-elastic material parameters were considered for both phases where: E_m_ = 3 GPa, υ_m_ = 0.36 and E_p_ = 70 GPa, υ_p_ = 0.22 are the Young’s moduli and Poisson ratios for epoxy matrix and glass particles, respectively. The FEA was performed by using ANSYS software. Figure 7b shows a visualization of the result of the DIC analysis based on the artificially deformed image of the speckle pattern in the Istra 4D software. Here, the distribution of strain ε_yy_ is visualized simultaneously with the accounted speckle pattern. Results of the DIC analysis are presented in detail in the further figures where obtained displacement (Figure 8) and strain distributions (Figure 9 and Figure 10) are compiled with the reference FE solution. In the case of visualization of the strain fields obtained by using FEM, a color scale was adjusted to the results of the DIC to provide a reliable comparison between the results. Moreover, the strain distributions related to the DIC solution and the reference FE solution are represented in histograms (Figure 11).

The displacement fields obtained by the DIC and the reference FE solution are in good agreement; the displacement fronts are distorted similarly due to the presence of reinforcing particles. The obtained strain distributions show that the created speckle pattern allows to expose the strain field heterogeneity and track the positions of the particles behind the speckle pattern. Nonetheless, the smoothing effect associated with the DIC, which is unavoidable at the boundary between the phases, is noticeable. Analysis of the histograms (Figure 11) leads to the conclusion that the widths of the strain distributions obtained by using both methods are similar. However, the smoothing effect is also visible—for the FE solution, the bimodal pattern is significantly noticeable, while the DIC results make the peaks of the histogram less pronounced. Especially for the strain ε_xx_, the bimodal character of the distribution is almost smoothed out. In conclusion, the proposed method of assessment of the speckle pattern made it possible to verify that the created speckle pattern is applicable for measuring the local strain fields for the expected size and shape of the reinforcing particles. Moreover, it shows a level of smoothing of the results due to the application of the DIC method accounting for the current speckle pattern.

### 3.2. Experimental versus Numerical Results

**Figure 12:**
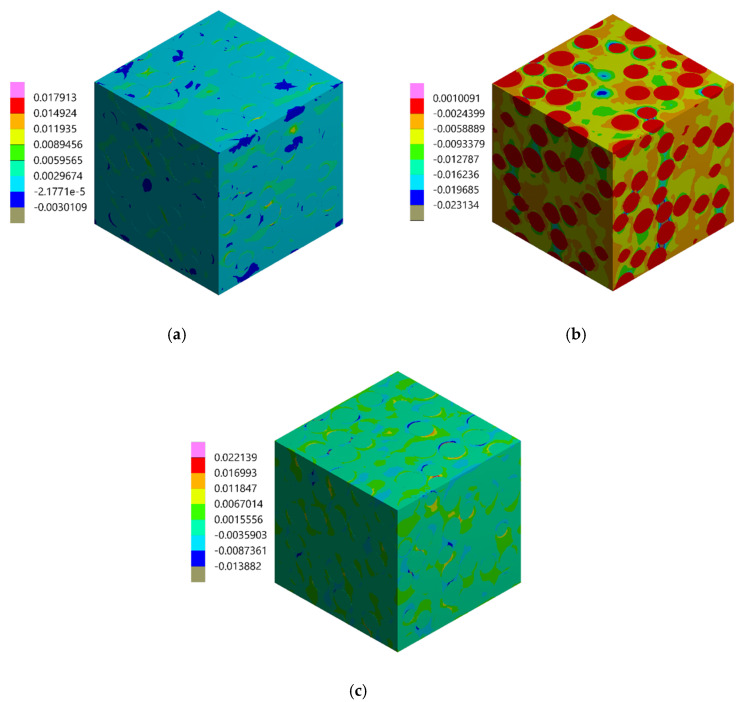
Strain distributions obtained by using FEM: (**a**) component ε_xx_, (**b**) component ε_yy_, (**c**) component ε_xy._

**Figure 13:**
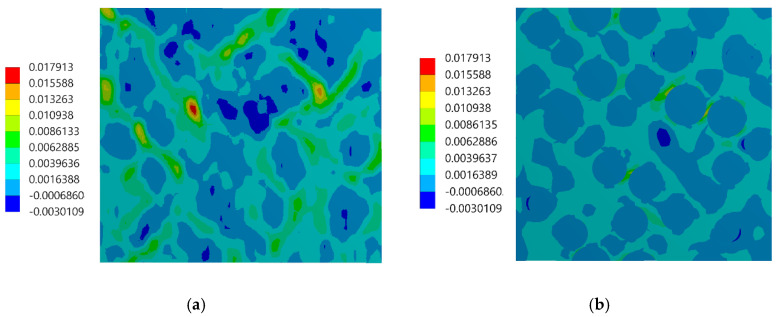
Strain ε_xx_ distributions: (**a**) experimental results (DIC), (**b**) numerical results (FEM).

**Figure 14:**
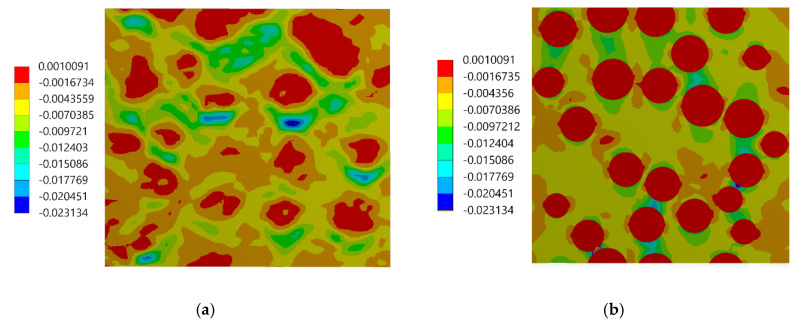
Strain ε_yy_ distributions: (**a**) experimental results (DIC), (**b**) numerical results (FEM).

**Figure 15:**
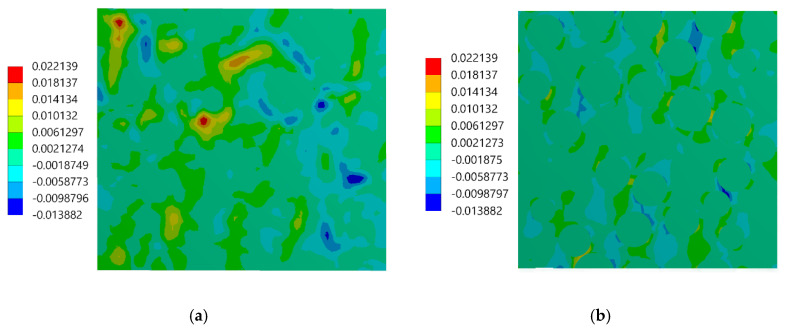
Strain ε_xy_ distributions: (**a**) experimental results (DIC), (**b**) numerical results (FEM).

**Figure 16:**
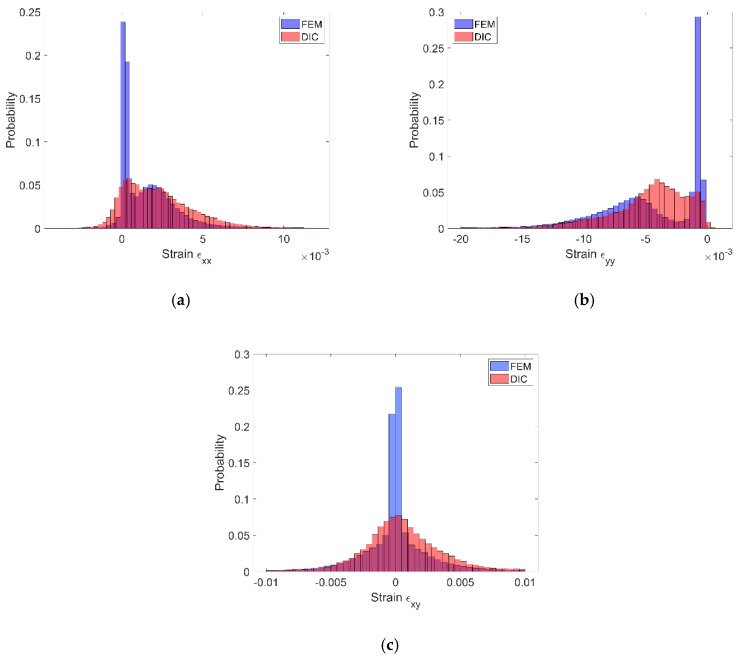
Histograms representing the strain distributions determined experimentally by DIC and numerically by FEM: (**a**) ε_xx_ component, (**b**) ε_yy_ component, (**c**) ε_xy_ component.

During the experimental test performed by using the DIC, two images were captured: the first one was associated with the undeformed state, the second one was related to the uniaxial compressive loading, the value of which was adjusted to not exceed the linear-elastic regime. Although the strain measurements were performed on the surface of the specimen, they were related to the composite, whose behavior must be modeled by using the three-dimensional representative volume element (RVE) to achieve consistency of results. Therefore, the FEA of the three-dimensional RVE was carried out by applying the periodic boundary conditions in a way to enforce the uniaxial compressive strain that matches the result of the DIC analysis. In other words, an average strain obtained from the DIC analysis was prescribed as the boundary condition of FEA; the value of the prescribed strain is ε = −0.004568. The same material parameters of the constituents as those provided in Section 3.1 were applied. The strain distributions computed by the FEM are presented in Figure 12; the color scale was adjusted to the results of the DIC to provide a reliable comparison between the results. Subsequently, in Figure 13, Figure 14 and Figure 15, the strain distributions obtained by the experimental test are complied with the results of the FEA (a view of one of the three-dimensional RVE’s faces is illustrated). Moreover, the histograms representing the strain distributions obtained by both methods are presented (Figure 16).

The experimentally and numerically determined strain distributions presented in Figure 13, Figure 14 and Figure 15 are in good agreement. Nonetheless, some discrepancies between the results are noticeable. The strain jumps between the phases are visible in the case of the numerical results, while they are smoothed out in the case of the experimental results. The peak values of the strains are higher in the case of the experimental results. The histograms presented in Figure 16 show that the widths of the distributions are similar in both cases. The histograms also illustrate that the distributions determined experimentally are smoothed out in comparison with the distributions obtained numerically. In the case of normal strains, a bimodal shape of the distributions, due to a strain partitioning between the phases, can be observed both for the experimental and numerical results. However, the bimodality is much more pronounced in the numerical case. The distributions of the shear strains have got almost symmetric unimodal shape. The discrepancies between the experimental and numerical results are slightly larger than the discrepancies between the results of the DIC analysis (which was conducted based on this same speckle pattern as the experimental test) and the reference “accurate” numerical solution (Section 3.1). Therefore, we conclude that the discrepancies between the experimental and numerical results depend not only on the errors associated with the usage of the DIC method, but also on the simplifications related to the numerical model (the same size and spherical shape of the reinforcing particles, the perfect interface between the phases, etc.). Nonetheless, it has to be pointed out that despite the simplifications the agreement between results is good. Consequently, usage of the representative volume elements simplifying the microstructural features, which are very often applied in multiscale computations, is justified and allows to obtain reasonable accuracy.

## 4. Conclusions

In this paper, both numerical and experimental investigation of the strain field at the level of the constituents of two-phase particle-reinforced composites were carried out. Moreover, usage of a novel approach for the assessment of the applicability of the speckle pattern to local strain field analysis was proposed by the digital image correlation method. The main findings from this work can be summarized as follows:

- The proposed method for the assessment of the speckle pattern made it possible to verify that the speckle pattern prepared on the surface of the specimen was applicable for measuring a local strain at the level of constituents of composite by using the DIC method. Moreover, it made it possible to predict how much the strain distributions obtained by the DIC were smoothed out by performing a comparison with the “accurate” reference solution.
- Good agreement between the local strain distributions determined experimentally by using the DIC and numerically by using FEA based on the RVE model has been noticed. The agreement between the results was good, despite the simplifications related to the geometrical model of the microstructure (RVE).
- The main difference in the full-field strains obtained experimentally and numerically was related to the region of the phase boundaries: the strain jumps between the phases are visible in the case of the numerical result, while they are smoothed out in the case of the experimental results.
- The normal elastic strain distributions had a bimodal character due to strain partitioning between the matrix and reinforcing particles. The bimodality has been revealed by both experimental and numerical results although the bimodality is much more pronounced in the numerical case. The shear strain distributions have got symmetric unimodal shape. The widths of the strain distributions are similar for both methods.

It has to be noted that the presented experimental results are based on the surface analysis, which gives limited information associated with the strain state. Moreover, the strain distributions may be different in the bulk of a solid compared to the values measured at the surface. To obtain three-dimensional strain fields, including measurement in the bulk, more advanced methods such as Digital Volume Correlation [44,45,46,47,48] or Raman spectroscopy [49,50,51,52,53] may be used.
